# Trends of the Global Burden of Disease Attributable to Cannabis Use Disorder in 204 Countries and Territories, 1990–2019: Results from the Disease Burden Study 2019

**DOI:** 10.1007/s11469-022-00999-4

**Published:** 2023-02-10

**Authors:** Heng Shao, Heyue Du, Quan Gan, Dequan Ye, Zhuangfei Chen, Yanqing Zhu, Shasha Zhu, Lang Qu, Junyan Lu, Yutong Li, Jing Duan, Yingqi Gu, Meiling Chen

**Affiliations:** 1 Department of Geriatrics, The First People’s Hospital of Yunnan Province, Yunnan, China; 2 The Affiliated Hospital of Kunming University of Science and Technology, Kunming, Yunnan China; 3 Department of Endocrinology and Metabolism, West China Hospital, Sichuan University, Chengdu, China; 4 Department of Nephrology, West China Hospital, Sichuan University, Chengdu, China; 5 Faculté de Médecine, Université Paris Saclay, Paris, France; 6 Department of Ultrasound in Medicine, the Second Affiliated Hospital, Zhejiang University School of Medicine, Hangzhou, China; 7 Medical Faculty, Kunming University of Science and Technology, Kunming, Yunnan China; 8 Department of Clinical Psychology, The First People’s Hospital of Yunnan Province, Yunnan, China; 9 West China School of Medicine, Sichuan University, Chengdu, China; 10 Institute of Medical Biology Chinese Academy of Medical Sciences, Kunming, China; 11 Department of Psychology, Mount Holyoke College, South Hadley, MA USA; 12 Yunnan Center for Disease Control and Prevention, Kunming, China

**Keywords:** Cannabis use disorder, Global burden of disease, Disability-adjusted life years, Age-standardized rate, Estimated annual percentage change

## Abstract

Cannabis is the fourth psychoactive substance to be legalized which are of far-reaching significance to the world. We analyzed data from the 2019 Global Burden of Disease Study (GBD) to estimate the incidence and prevalence of cannabis use disorder (CUD) and calculated the disease burden of CUD in 204 countries and territories and 21 regions over the past three decades. We reported disease burden due to CUD in terms of disability-adjusted life years (DALYs), age-standardized rate (ASR), estimated annual percentage change (EAPC), and analyzed associations between the burden of CUD and sociodemographic index (SDI) quintiles. Globally, the number of incidence cases of CUD was estimated to be increasing by 32.3% from 1990 to 2019 and males are nearly double higher than that of female. DALYs increase 38.6% from 1990. Young people aged 20–24 years old with cannabis use disorder have the highest DALYs in 2019, followed by those younger than 20 years old. India, Canada, USA, Qatar, Kenya, and high SDI quintile areas showed a high burden of disease. Nearly 200 million individuals are cannabis users worldwide, and CUD is a notable condition of GBD. The global cultivation of cannabis, rooted in different cultures, diversified access to cannabis, legalization in controversy, the promotion of medical cannabis, and many other factors promote the global cannabis industry is constantly updated and upgraded. It deserves more discussion in the future in terms of pathophysiological mechanisms, socioeconomics, law, and policy improvement.

**Supplementary Information:**

The online version contains supplementary material available at 10.1007/s11469-022-00999-4.

## Introduction

According to a report (United Nations Office on Drugs & Crime, 2020c) released by the United Nations Office on Drugs and Crime (UNODC) in the year 2020, more than a quarter of 1 billion people in the world use drugs, over 35 million people are affected by drug use disorders, and cannabis is still the most commonly used drug by far. Unlike other plant-based drugs that are grown and produced only in a few countries, cannabis is produced in almost all countries in the world. During 2010–2018, 151 countries reported the cultivation of cannabis plants, of which the population accounted for 96% of the global population (United Nations Office on Drugs & Crime, 2020b). The usage of cannabis can be divided into medical cannabis and recreational cannabis; however, this is a controversial issue still (Bostwick, 2012). Non-medical cannabis is illegal in most parts of the world. But so far, 37 states of the USA, Canada, and Uruguay where these jurisdictions were the first to legalize a commercial market of cannabis for recreational purposes (Cannabis Laws and Regulations of Canada, n.d.; Marihuana y Sus Derivados, n.d.; U.S. State Medical Cannabis Laws, n.d.).

Cannabis is referred to as the cannabis plant material or its extracts that contain substantial amounts of Δ9-tetrahydrocannabinol (THC). THC can produce a desire for repeated use, which in some users develop into cannabis use disorder (CUD) (Connor et al., 2021). CUD may lead to a series of withdrawal syndromes, such as irritability, aggression, anxiety, sleep disorder, and restlessness. In addition, long-term use of cannabis seems to involve underlying neurophysiological changes in reward, stress, and executive function circuits (Zehra et al., 2018). At present, cannabis is the fourth psychoactive substance to be legalized after alcohol, tobacco, and coffee which are of far-reaching significance to the world (Degenhardt & Hall, 2012). It seems to increase the frequency and side effect of cannabis use, which leads to many adverse effects on public health (Hall et al., 2019; Hall & Lynskey, 2020).

From 1990 to 2019, the burden of substance use disorders has increased, including alcohol, amphetamines, cannabis, and cocaine as substance use disorders (Degenhardt et al., 2018; Pan et al., 2020; Whiteford et al., 2013). In this research, we analyzed data from the 2019 Global Burden of Disease Study (GBD) to estimate the incidence and prevalence of CUD and calculated the disease burden of CUD in 204 countries and territories and 21 regions over the past three decades. We reported disease burden due to CUD in terms of disability-adjusted life years (DALYs), age-standardized rate (ASR), estimated annual percentage change (EAPC), and analyzed associations between CUD burden and sociodemographic index (SDI) quintiles.

## Method

### Definition of GBD Epidemiological Parameters

CUD is defined as habitual cannabis use, cravings, and inability to reduce or stop cannabis use despite experienced physical and/or psychological harm (Segal, 2010; World Health Organization, 1992). For the global disease burden, the diagnostic criteria for CUD were in accordance with the DSM-IV and ICD-10 (Vos et al., 2020). The age-standardized incidence rate (ASIR), age-standardized prevalence rate (ASPR), age-standardized rate of DALYs (ASDR), and EAPC were used to quantify the incidence trends of CUD.

DALYs is a measure of overall burden of a certain type of disease since the 90th, expressed as the cumulative number of years lost due to ill-health, disability, or early death. They are calculated by summing the number of years of life lost due to premature mortality (YLLs) and the number of years of healthy life lost due to disability (YLDs) (World Health Organization, n.d.). The crude rates, including incidence, prevalence, and DALYs, are extracted by the occurrence of a certain disease in a whole population of one country which could affected by its age distribution. Age-standardized rates are used to compare rates of health outcome (e.g., disease incidence, prevalence, DALYs, or other health-related events) between different populations. To calculate ASR, the crude rate (the outcome rate observed in the population) is adjusted for the age structure of the population in each country and region.

EAPC is a summary and widely used measure of the ASIR, ASPR, and ASDR trends over a specific time period. To take ASIR as an example, a regression line was fitted to the natural logarithm of the ASIR values, which can be represented as y = α + βx + ϵ, where y = ln (ASIR) and x = calendar year. The EAPC was calculated as 100 × (exp (β) − 1), and its 95% confidence interval (CI) was extracted from a linear regression model (Liu et al., 2020).

### Data Source and GBD Estimation Method

The GBD of CUD estimation process including case definition, input data, age and sex splitting, data adjustment, and modeling strategy is clearly defined in the appendix 1 of systematic analysis for the Global Burden of Disease Study 2019 (Vos et al., 2020). Sources for the disease burden data of CUD can be explored using the online GHDx (Global Health Data Exchange) data source query tool (https://vizhub.healthdata.org/gbd-results/). We obtained incidence number, prevalence number, DALYs, ASIR, ASPR, and ASDR of CUD from 1990 to 2019 according to sex, 21 regions, and 204 countries and territories. Based on the SDI which combines information about the economy, education, and fertility rate of countries around the world, as a representation of social and economic development present in 2019, the 204 countries or territories were divided into five quintiles: low, low-middle, middle, high-middle, and high. The general methods used in GBD 2019 are described in detail on the official website (http://www.healthdata.org/gbd/2019), which also provides the SDI values from 1990 to 2019 at the global, regional, and national levels. To analyze global trends, we also assessed the trends of CUD according to the following age stratification used in GBD 2019: < 20, 20–24, 25–29, 30–34, 35–39, 40–44, 45–49, 50–54, 55–59, 60–64, 65–69, 70–74, 75–79, and 80 plus years old which will generally be reflecting adult CUD, for adolescent combined into < 20 group.

Incidence, prevalence, DALYs, ASIR, ASPR, and ASDR were extracted from the GBD database. EAPC was manually estimated based on its definition. In this article, we highlight the absolute value of incidence and prevalence, the variation of ASIR, ASPR, and ASDR stratified by SDI over time, as well as the EAPC value of three parameters above to be reported by splitting value into five intervals that based quintiles value. Moreover, informed consent for accessing the GBD data was waived by the University of Washington Institutional Review Board. This study followed the Guidelines for Accurate and Transparent Health Estimates Reporting (GATHER) (Stevens et al., 2016). All statistical processes were conducted in R (version 4.0.3).

## Results

### The Burden of CUD at the Global Level

Globally, the number of incidence and prevalence cases of CUD was estimated to be increasing gradually, by 32.3% and 38.6% in 2019. The global DALYs of CUD in 2019 summed up 0.69 million indicate an increase of 38.6% from 1990. However, the ASIR and ASPR are relatively stable, from 48.2/100,000 persons (95% *CI* = 37.0 to 65.8/100,000 persons) in 1990 to 48.8/100,000 persons (95% *CI* = 37.1 to 65.8/100,000 persons) in 2019 and 303.3/100,000 persons (95% *CI* = 227.3 to 396.9/100,000persons) in 1990 to 303.4/100,000 persons (95% *CI* = 226.1 to 396.2/100,000 persons) in 2019, respectively. The ASDR was almost unchanged (Tables 1 and 2).

**Table 1:** The incident cases and prevalence and their age-standardized rate of cannabis use disorder in 1990 and 2019, and their temporal trends from 1990 to 2019

	**Incidence**						**Prevalence**					
	1990 incidence No. × 10^3^ (95% *CI*)	1990 age-standardized incidence rate per 100,000 No. (95% *CI*)	2019 Incidence No. × 10^3^ (95% *CI*)	2019 age-standardized incidence rate per 100,000 No. (95% *CI*)	percentage change in age-standardized incidence rate, 1990–2019 (95% *CI*)	EAPC 1990–2019 (95% *CI*)	1990 prevalence No. × 10^3^ (95% *CI*)	1990 age-standardized prevalence rate per 100,000 No. (95% *CI*)	2019 prevalence No. × 10^3^ (95% *CI*)	2019 age-standardized prevalence rate per 100,000 No. (95% *CI*)	Percentage change in age-standardized prevalence rate, 1990–2019 (95% *CI*)	EAPC 1990–2019 (95% *CI*)
Global	2825.09 (2140.03, 3903.21)	48.2 (37, 65.8)	3737.24 (2847.47, 5041.04)	48.8 (37.1, 65.8)	2.7% (0%, 6.1%)	− 0.01% (− 0.08%, 0.07%)	17,199.34 (12,785.70, 22,801.80)	303.3 (227.3, 396.9)	23,845.50 (17,836.83, 30,900.97)	303.4 (226.1, 396.2)	1.1% (− 1.5%, 4.3%)	− 0.06% (− 0.14%, 0.02%)
***Sex***												
Male	1785.66 (1341.79, 2470.04)	60.1 (45.8, 81.9)	2421.04 (1842.34, 3275.28)	62.1 (47.1, 84.1)	4.7% (1.4%, 9.1%)	0.07% (− 0.02%, 0.15%)	10,975.93 (8169.48, 14,529.60)	383.1 (287.7, 501.6)	15,628.85 (11,741.82, 20,364.54)	393.4 (295, 513.8)	4.1% (0.9%, 8%)	0.03135305% (− 0.06%, 0.12%)
Female	1039.42 (786.25, 1433.11)	36.1 (27.7, 49.1)	1316.20 (1011.69, 1761.55)	34.9 (26.7, 46.6)	− 1.2% (− 4.2%, 1.9%)	− 0.14% (− 0.20%, − 0.09%)	6223.41 (4608.85, 8254.87)	221.9 (166.7, 291.1)	8216.66 (6149.92, 10,670.39)	211.4 (157.1, 275)	− 4% (− 6.9%, − 1.2%)	− 0.2175315% (− 0.29%, − 0.15%)
***Socio-demographic index***												
High SDI	819.84 (640.39, 1123.27)	108.6 (84.2, 149.7)	817.29 (640.15, 1098.61)	107.9 (83.6, 146.5)	− 0.6% (− 4%, 2.8%)	0.08% (0.05%, 0.11%)	5753.04 (4467.36, 7377.38)	697.1 (543.6, 900.3)	5886.72 (4642.03, 7454.26)	684.4 (535, 882.2)	− 0.7% (− 3.9%, 2.5%)	0.04% (0%, 0.07%)
High-middle SDI	567.28 (431.86, 774.06)	46.8 (36, 64.3)	580.49 (456.87, 763.51)	48.5 (37.5, 65.3)	5.4% (1.7%, 9.5%)	0.05% (− 0.09%, 0.19%)	3497.07 (2610.02, 4591.38)	281.7 (212.5, 368.6)	3970.15 (3077.99, 4999.35)	293 (223.5, 378.1)	5.7% (1.8%, 10.2%)	− 0.01% (− 0.14%, 0.12%)
Middle SDI	729.38 (535.23, 1023.51)	36.7 (27.7, 49.9)	973.57 (739.32, 1320.85)	41.1 (30.9, 55.9)	15.3% (11.9%, 19%)	0.43% (0.33%, 0.54%)	4116.086 (2935.30, 5608.26)	218.7 (159.3, 289.5)	6400.63 (4733.06, 8408.22)	256.9 (188.1, 338.4)	19.5% (15.9%, 23.6%)	0.38% (0.26%, 0.49%)
Low-middle SDI	497.84 (364.41, 708.11)	41 (30.8, 56.7)	844.96 (624.63, 1190.11)	43.4 (32.6, 60.7)	13.4% (8.7%, 17.6%)	0.13% (0.04%, 0.23%)	2704.97 (1926.50, 3650.08)	243.3 (178.8, 321)	4976.55 (3645.20, 6656.179)	263.3 (195.4, 348.6)	9.8% (5.4%, 14.2%)	0.18% (0.07%, 0.29%)
Low SDI	208.77 (150.72, 307.54)	37.9 (28.2, 52.9)	448.29 (322.94, 650.59)	35.5 (26.5, 49.6)	3.4% (0.5%, 6%)	0.01% (− 0.07%, 0.08%)	1116.13 (784.62, 1563.36)	227.4 (164.9, 308.3)	2594.81 (1810.30, 3650.36)	230.2 (167.3, 311.5)	2.5% (− 0.1%, 5.1%)	0.03% (0%, 0.06%)
***Region***												
Andean Latin America	17.29 (12.61, 23.76)	40 (29.9, 53.9)	26.64 (19.98, 35.72)	40.1 (30.1, 53.6)	− 3% (− 9.6%, 2.6%)	0.06% (0.04%, 0.08%)	97.43 (68.29, 131.69)	245.2 (176.6, 326.7)	163.80 (119.36, 215.07)	244.9 (180.1, 319.1)	1.5% (− 5.8%, 7.8%)	0.05% (0.03%, 0.08%)
Australasia	35.87 (27.79, 46.70)	181.2 (141.3, 234.7)	29.10 (22.63, 37.36)	127.8 (98.6, 165.5)	− 29.9% (− 38.5%, − 21.3%)	− 0.8% (− 0.96%, − 0.65%)	258.22 (213.70, 309.80)	1231 (1015.1, 1478.9)	205.48 (168.97, 250.28)	814.6 (664.7, 995.3)	− 32.8% (− 37.8%, − 26.8%)	− 0.81% (− 1.01%, − 0.62%)
Caribbean	27.84 (19.71, 41.63)	69.4 (50.2, 102)	32.27 (23.24, 47.18)	69.4 (49.9, 102)	− 3.6% (− 7%, − 0.2%)	− 0.12% (− 0.15%, − 0.09%)	175.45 (116.00, 260.77)	449.7 (306.8, 650.6)	215.17 (146.88, 311.34)	447.6 (303.8, 649.9)	0% (− 3.1%, 3.5%)	− 0.16% (− 0.2%, − 0.12%)
Central Asia	23.68 (15.99, 36.49)	31.5 (21.9, 47.4)	30.06 (21.12, 44.60)	32.4 (22.5, 49)	1.4% (− 0.8%, 3.9%)	0.09% (0.08%, 0.1%)	137.63 (88.75, 208.79)	188.6 (124.9, 279.4)	188.76 (124.42, 276.74)	195.5 (127.9, 288.6)	5.3% (3.9%, 7%)	0.12% (0.11%, 0.13%)
Central Europe	69.76 (52.65, 96.59)	59.5 (44.4, 82.2)	44.61 (35.20, 57.68)	55.1 (42.7, 73.7)	− 2.7% (− 11%, 4.9%)	− 0.11% (− 0.17%, − 0.05%)	432.47 (317.52, 582.16)	365.2 (266, 497.4)	317.58 (248.69, 400.93)	347.5 (268.6, 446.7)	− 3.1% (− 9.9%, 5.2%)	− 0.03% (− 0.09%, 0.03%)
Central Latin America	69.38 (51.76, 97.73)	36.6 (28, 50)	108.69 (84.95, 143.55)	41.5 (32.6, 54.6)	21.6% (12.9%, 31.1%)	0.28% (0.19%, 0.37%)	392.72 (286.11, 538.62)	226.1 (169.5, 301.4)	709.85 (565.89, 885.92)	267.2 (213.6, 332.8)	21.1% (11.6%, 33.2%)	0.33% (0.22%, 0.44%)
Central Sub-Saharan Africa	18.06 (12.29, 27.43)	30.8 (22, 45.4)	45.17 (30.82, 68.39)	30.9 (22, 45.5)	− 1.6% (− 3.2%, 0.1%)	0% (0%, 0%)	96.27 (61.76, 142.93)	183.6 (125.2, 261.4)	241.67 (156.10, 356.11)	184.2 (126, 262)	1.1% (0.9%, 1.4%)	0.01% (0.01%, 0.01%)
East Asia	436.06 (313.21, 616.49)	29.8 (22.1, 41.1)	465.26 (355.70, 633.73)	36.5 (26.7, 51.8)	23.7% (16.2%, 32%)	0.41% (0.18%, 0.63%)	2418.61 (1715.54, 3269.30)	170.7 (124.6, 225.4)	3104.54 (2325.35, 4054.64)	211.1 (150.9, 282.9)	27% (18.8%, 34.8%)	0.42% (0.18%, 0.66%)
Eastern Europe	106.50 (78.08, 153.40)	52.5 (37.8, 77)	84.19 (63.69, 119.70)	55.7 (40.2, 81.7)	6% (− 0.5%, 13.2%)	0.09% (0.01%, 0.18%)	701.02 (493.19, 975.70)	325.2 (225.4, 456.5)	585.64 (422.34, 792.29)	344.5 (236.8, 492.4)	7.4% (1.2%, 15.4%)	0.1% (0.01%, 0.18%)
Eastern Sub-Saharan Africa	82.92 (58.22, 124.06)	38.6 (28.3, 55.3)	187.02 (131.00, 279.24)	38.1 (27.8, 55.3)	3.7% (− 1.7%, 7.4%)	0.09% (0.04%, 0.14%)	439.67 (300.22, 645.46)	234.7 (167.3, 326.9)	1004.54 (674.61, 1496.13)	229.5 (162.2, 325.4)	− 1% (− 5.9%, 2.2%)	0.07% (0.01%, 0.12%)
High-income Asia Pacific	139.33 (99.22, 202.38)	78.2 (55.5, 114.2)	99.99 (73.51, 140.98)	77.2 (54.2, 112.1)	− 0.3% (− 3.8%, 3.1%)	− 0.03% (− 0.04%, − 0.02%)	862.30 (602.03, 1191.39)	474.1 (327.1, 659.8)	698.29 (510.66, 931.83)	469.1 (324.6, 654.9)	0.9% (− 3%, 4.3%)	− 0.01% (− 0.02%, 0%)
High-income North America	385.22 (297.94, 531.79)	153.8 (117.9, 212.4)	442.55 (343.31, 602.35)	149.3 (114.7, 203.8)	− 2.7% (− 7.8%, 2.9%)	0% (− 0.04%, 0.04%)	2884.18 (2222.61, 3719.74)	1033 (790.6, 1348.5)	3233.50 (2535.39, 4141.75)	998.2 (773.9, 1301.8)	− 2.5% (− 7.6%, 2.6%)	− 0.02% (− 0.07%, 0.03%)
North Africa and Middle East	79.53 (56.36, 117.78)	20.6 (15.1, 29.5)	148.36 (108.30, 212.29)	22.9 (16.7, 32.9)	6.9% (1.9%, 11.4%)	0.29% (0.24%, 0.34%)	423.52 (285.89, 622.43)	120.1 (84.3, 169.1)	893.95 (627.44, 1265.22)	136.6 (96.4, 193.3)	14.8% (10.1%, 19.4%)	0.36% (0.31%, 0.41%)
Oceania	5.69 (3.81, 9.14)	74.3 (51.6, 114.6)	11.15 (7.67, 17.28)	75 (52.7, 115.1)	− 3.3% (− 6.9%, 0.6%)	0.02% (0.01%, 0.03%)	33.39 (21.15, 52.74)	473.7 (309.9, 722.6)	68.50 (44.64, 104.91)	477.1 (316.7, 718.5)	1.3% (− 1%, 4%)	0.01% (0%, 0.02%)
South Asia	526.37 (385.17, 757.38)	45.6 (34.2, 63.7)	1039.61 (765.76, 1462.18)	51.5 (38.2, 71.7)	23.4% (15.7%, 29.7%)	0.31% (0.18%, 0.44%)	2881.73 (2068.32, 3883.92)	270.7 (201.3, 354.9)	6097.13 (4469.55, 8226.54)	311.7 (232.3, 415.3)	16.7% (9.8%, 23.9%)	0.36% (0.21%, 0.51%)
Southeast Asia	234.39 (168.24, 341.70)	43.6 (32, 62)	317.59 (234.19, 448.05)	45.4 (33.3, 65.1)	6.6% (3.3%, 9.9%)	0.09% (0.05%, 0.13%)	1313.15 (893.30, 1884.32)	259.8 (184, 361.3)	1941.62 (1382.28, 2701.11)	271.5 (191.3, 379.7)	6.9% (3.7%, 10.4%)	0.11% (0.07%, 0.14%)
Southern Latin America	25.22 (19.51, 33.00)	48.6 (37.8, 63.6)	34.88 (28.42, 42.65)	56.4 (46.1, 68.5)	15% (1.1%, 30%)	0.46% (0.38%, 0.55%)	146.82 (116.60, 183.49)	288.8 (230.3, 360.1)	235.85 (207.48, 268.56)	357.9 (314.6, 408.3)	25.9% (10.8%, 43.9%)	0.64% (0.56%, 0.71%)
Southern Sub-Saharan Africa	25.44 (18.49, 37.16)	41.2 (30.9, 58.4)	33.03 (24.86, 46.44)	39.1 (29.5, 54.9)	− 2.9% (− 8.8%, 6.9%)	− 0.03% (− 0.09%, 0.04%)	145.17 (103.39, 203.88)	256.1 (187.2, 346.5)	205.90 (150.04, 280.08)	244 (180.3, 329.8)	− 2.9% (− 8.9%, 4.3%)	− 0.01% (− 0.07%, 0.05%)
Tropical Latin America	129.40 (92.14, 192.97)	73.3 (53.6, 107.3)	155.61 (117.88, 210.22)	70.7 (53.1, 97.4)	0.7% (− 12.4%, 13.2%)	− 0.17% (− 0.21%, − 0.13%)	820.08 (570.35, 1162.34)	494.8 (352.9, 684.6)	1101.16 (810.24, 1439.78)	472 (344.5, 623.5)	− 3.5% (− 14%, 7.7%)	− 0.3% (− 0.38%, − 0.22%)
Western Europe	344.77 (273.68, 449.16)	104.3 (81.9, 137.8)	292.09 (236.58, 375.67)	95.4 (76.7, 123.9)	− 8.5% (− 14.5%, − 1.7%)	− 0.09% (− 0.17%, 0%)	2315.80 (1830.07, 2914.67)	627.4 (494.6, 797.6)	2058.21 (1696.63, 2479.43)	600.9 (493.6, 739.9)	− 3.3% (− 9.4%, 2.7%)	0.02% (− 0.07%, 0.11%)
Western Sub-Saharan Africa	42.33 (29.88, 61.30)	21.5 (15.8, 30.3)	109.35 (77.23, 158.81)	21.9 (16.2, 30.7)	3.9% (0.3%, 7.2%)	0.02% (− 0.01%, 0.05%)	223.68 (154.59, 316.40)	126.1 (90, 171.4)	574.36 (396.61, 814.03)	128.1 (92, 174.5)	2.6% (− 0.8%, 5.8%)	0.01% (− 0.02%, 0.04%)

*CI*, confidence interval; *EAPC*, estimated annual percentage change; age-stratified population estimations of countries and regions around the world are presented in link (https://ghdx.healthdata.org/record/ihme-data/gbd-2019-population-estimates-1950-2019) here that could be used as an important reference for incidence number and prevalence number

**Table 2:** The DALYs and their age-standardized rate of cannabis use disorder in 1990 and 2019, and their temporal trends from 1990 to 2019

	**DALY**					
	1990 DALYs No. × 10^3^ (95% *CI*)	1990 age-standardized DALYs rate per 100,000 No. (95% *CI*)	2019 DALYs No. × 10^3^ (95% *CI*)	2019 age-standardized DALYs rate per 100,000 No. (95% *CI*)	Percentage change in all age DALYs, 1990–2019 (95% *CI*)	EAPC 1990–2019 (95% *CI*)
Global	498.05 (297.58, 780.58)	8.8 (5.3, 13.7)	690.34 (420.82, 1077.12)	8.8 (5.3, 13.7)	41.5% (32%, 53.9%)	− 0.05% (− 0.13%, 0.03%)
***Sex***						
Male	318.89 (189.59, 500.54)	11.1 (6.7, 17.2)	453.79 (275.65, 707.59)	11.4 (6.9, 17.8)	45.1% (34.2%, 57.8%)	0.04% (− 0.05%, 0.13%)
Female	179.16 (107.44, 282.35)	6.4 (3.9, 9.9)	236.55 (142.53, 369.27)	6.1 (3.7, 9.5)	35% (25.2%, 47.5%)	− 0.21% (− 0.28%, − 0.14%)
***Socio-demographic index***						
High SDI	166.33 (100.32, 254.45)	20.2 (12.1, 31)	169.59 (105.19, 258.61)	19.8 (12.1, 30.4)	− 14.4% (− 18.5%, − 9.7%)	0.03% (0%, 0.06%)
High-middle SDI	101.62 (61.14, 158.61)	8.2 (5, 12.8)	115.41 (70.86, 179.50)	8.5 (5.2, 13.1)	8.4% (− 0.7%, 20.1%)	0% (− 0.13%, 0.13%)
Middle SDI	119.63 (69.18, 191.78)	6.3 (3.8, 10)	185.84 (112.32, 292.23)	7.5 (4.5, 11.7)	52.2% (39%, 69%)	0.39% (0.28%, 0.5%)
Low-middle SDI	77.98 (44.61, 124.77)	7 (4.1, 11)	143.82 (85.07, 223.88)	7.6 (4.5, 11.8)	119.7% (100.1%, 144.4%)	0.2% (0.1%, 0.31%)
Low SDI	32.14 (18.16, 52.15)	6.5 (3.8, 10.4)	75.19 (43.00, 122.34)	6.7 (3.9, 10.6)	141.4% (112.1%, 170.2%)	0.06% (0.02%, 0.09%)
***Region***						
Andean Latin America	2.84 (1.61, 4.54)	7.1 (4.1, 11.2)	4.76 (2.76, 7.50)	7.1 (4.2, 11.2)	79.1% (46.7%, 123.9%)	0.06% (0.04%, 0.09%)
Australasia	7.45 (4.65, 11.00)	35.5 (22.3, 52.4)	5.92 (3.67, 8.79)	23.5 (14.6, 34.8)	− 39.2% (− 45.5%, − 31.9%)	− 0.84% (− 1.03%, − 0.65%)
Caribbean	5.10 (2.81, 8.70)	13.1 (7.3, 21.9)	6.24 (3.51, 10.50)	13 (7.3, 22)	8.1% (− 4.1%, 24.2%)	− 0.16% (− 0.2%, − 0.12%)
Central Asia	4.02 (2.16, 7.04)	5.5 (3, 9.5)	5.52 (3.05, 9.44)	5.7 (3.1, 9.8)	33.6% (18.2%, 54.5%)	0.12% (0.11%, 0.13%)
Central Europe	12.60 (7.34, 19.92)	10.6 (6.2, 16.9)	9.25 (5.65, 14.27)	10.1 (6.1, 15.7)	− 16.2% (− 26%, − 3.4%)	− 0.03% (− 0.09%, 0.03%)
Central Latin America	11.4 (6.61, 18.27)	6.6 (3.9, 10.4)	20.66 (12.77, 31.10)	7.8 (4.8, 11.7)	48.1% (26.4%, 78.3%)	0.35% (0.24%, 0.46%)
Central Sub-Saharan Africa	2.77 (1.51, 4.63)	5.3 (3, 8.7)	7.01 (3.75, 11.93)	5.3 (3, 8.8)	156% (113.4%, 209.1%)	0.04% (0.03%, 0.05%)
East Asia	70.59 (40.85, 111.90)	5 (2.9, 7.8)	90.60 (53.51, 140.32)	6.2 (3.5, 9.6)	37.7% (19.3%, 61.1%)	0.44% (0.2%, 0.68%)
Eastern Europe	20.36 (11.78, 32.70)	9.5 (5.3, 15.6)	17.04 (10.05, 27.25)	10.1 (5.7, 16.8)	− 13.1% (− 20.7%, − 3.8%)	0.11% (0.02%, 0.19%)
Eastern Sub-Saharan Africa	12.71 (6.97, 21.30)	6.8 (3.8, 11)	29.21 (15.89, 49.39)	6.7 (3.8, 10.9)	171.9% (135.9%, 207.3%)	0.09% (0.03%, 0.15%)
High-income Asia Pacific	25.04 (14.41, 40.32)	13.8 (7.8, 22.3)	20.29 (11.95, 31.65)	13.7 (7.7, 22.1)	− 30.2% (− 35.7%, − 23.6%)	− 0.01% (− 0.02%, 0%)
High-income North America	83.10 (50.03, 127.41)	29.8 (17.7, 46)	92.65 (57.11, 141.80)	28.7 (17.4, 44)	− 16.4% (− 21.2%, − 11.3%)	− 0.03% (− 0.08%, 0.02%)
North Africa and Middle East	12.41 (6.70, 21.40)	3.5 (2, 5.8)	26.17 (14.67, 43.61)	4 (2.3, 6.6)	113.7% (89.6%, 143.8%)	0.36% (0.31%, 0.41%)
Oceania	0.96 (0.51, 1.72)	13.7 (7.4, 23.8)	1.99 (1.06, 3.43)	13.8 (7.5, 23.7)	14.3% (− 6%, 40.2%)	0.02% (0.01%, 0.03%)
South Asia	82.72 (47.47, 131.26)	7.8 (4.6, 12.1)	175.62 (101.69, 273.03)	9 (5.2, 13.9)	144.5% (119.8%, 174.1%)	0.38% (0.23%, 0.54%)
Southeast Asia	38.11 (21.31, 62.81)	7.5 (4.3, 12.3)	56.46 (33.18, 90.40)	7.9 (4.6, 12.7)	53.1% (38.7%, 71.4%)	0.12% (0.09%, 0.16%)
Southern Latin America	4.28 (2.60, 6.44)	8.4 (5.1, 12.7)	6.86 (4.44, 10.07)	10.4 (6.7, 15.2)	34.3% (13.6%, 63%)	0.66% (0.58%, 0.73%)
Southern Sub-Saharan Africa	4.22 (2.38, 7.07)	7.4 (4.3, 12)	5.97 (3.48, 9.50)	7.1 (4.2, 11.1)	− 2.4% (− 14.7%, 12.3%)	− 0.01% (− 0.07%, 0.04%)
Tropical Latin America	23.67 (13.48, 38.83)	14.3 (8.3, 23.1)	31.76 (18.83, 49.29)	13.6 (8.1, 21.3)	21.5% (4.3%, 42.8%)	− 0.29% (− 0.37%, − 0.21%)
Western Europe	67.16 (41.28, 101.61)	18.2 (11.2, 27.6)	59.62 (37.77, 89.01)	17.4 (10.9, 26.1)	− 13.3% (− 19.6%, − 5.9%)	0.02% (− 0.07%, 0.1%)
Western Sub-Saharan Africa	6.48 (3.70, 10.73)	3.7 (2.1, 5.8)	16.73 (9.37, 27.38)	3.7 (2.2, 5.9)	117.5% (87.5%, 149.8%)	0.03% (0%, 0.06%)

*DALYs*, disability-adjusted life years; *CI*, confidence interval; *EAPC*, estimated annual percentage change

Of note, the number of male incidence and prevalence cases exceeds that of female cases in any year. Also, the number of DALYs for male cases was much higher than that for female cases in every year. It demonstrated 0.45 million (95% *CI* = 0.28 to 0.71 million) for males and 0.24 million (95% *CI* = 0.14 to 0.37 million) for females in 2019, respectively. What can be clearly seen is the continual growth of ASIR and ASPR in male and that steady decline in female. The ASDR for males increased slightly and decreased in female, EAPC revealed 0.04% (95% *CI* =  − 0.05–0.13%) and − 0.21% (95% *CI* =  − 0.28–0.14%), respectively. The ASDR was much higher for males than for females in any year: in 2019; it was 11.4/100,000 persons (95% *CI* = 6.9 to 17.8/100,000 persons) for males and 6.1/100,000 persons (95% *CI* = 3.7 to 9.5/100,000 persons) for female (Table 2).

**Fig. 1:**
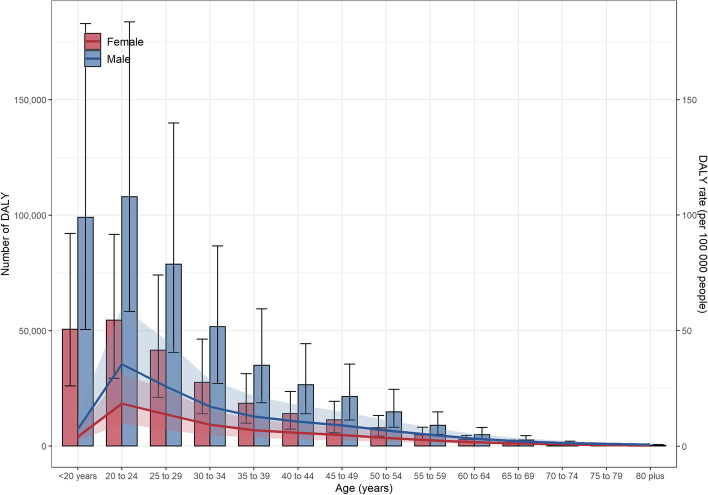
The distribution of DALYs caused by CUD by age and gender. DALYs, Disability-Adjusted Life Years; CUD, Cannabis Use Disorder

According to the age group, DALYs peaked at 20–24 years old and the second highest was the subgroup younger than 20 years old in 2019. In these two subgroups, males have a much higher than females (Fig. 1).

### The Burden of CUD at the National Level

Among the 204 countries and territories, those data were analyzed, the five largest number of incidence and prevalence cases in 2019 were India, China, USA, Brazil, and Indonesia. Compared to 1990, Indonesia replaced Japan entered to the top fifth. Countries with the lowest number of incidence and prevalence cases were Tokelau, Niue, Nauru, Tuvalu, and Palau (Supplementary Tables 1A and 1B).

From 1990 to 2019, the number of incidence cases sharply increased in Qatar and Equatorial Guinea at 538.7% and 352.5%. And prevalence cases that increased the most were in Qatar and the United Arab Emirates, at 656.3% and 357.3%, respectively. For incidence cases, India had the largest increase rate (100.9%) and China increased slightly (6.9%) among the five most populous countries. An obvious reduction in the number of incidence cases was in Bosnia and Herzegovina (− 54.2%) and Italy (− 47.0%) (Supplementary Table 1A). In terms of prevalence cases rate, India increased 116.0% and China increased gradually at 28.9%. Bosnia and Herzegovina declined 53.1% and Italy dropped 48.1% (Supplementary Table 1B). The five countries with the highest numbers of DALYs in 2019 were India, China, USA, Brazil, and Indonesia. Compared with 1990, the increase of DALYs was largest in Qatar and Equatorial Guinea, at 653.3% and 362.2%. Among the five most populous countries, the increase of DALYs was largest in India (116.8%) and all the five countries were increased. The number of DALYs reduced most significantly in Bosnia and Herzegovina (− 53.03%) and Italy (− 48.10%) (Supplementary Table 1C).

In 2019, ASIR and ASDR were the highest in Canada (180.5/100,000 persons, 95% *CI* = 157.1 to 208.6) and (35.1/100,000 persons, 95% *CI* = 22.7 to 51.0), followed by the USA (146.5/100,000 persons, 95% *CI* = 109.8 to 204.6) and (28.1/100,000 persons, 95% *CI* = 16.7 to 43.9), respectively. The lowest ASIR and ASDR were Turkey (15.3/100,000 persons, 95% *CI* = 11.4 to 20.9) and (2.5/100,000 persons, 95% *CI* = 1.4 to 4.1), followed by the Togo (17.5/100,000 persons, 95% *CI* = 12.7 to 24.4) and (2.8/100,000 persons, 95% *CI* = 1.6 to 4.6), respectively. ASPR shares the similar trend with ASIR and ASDR.

**Fig. 2:**
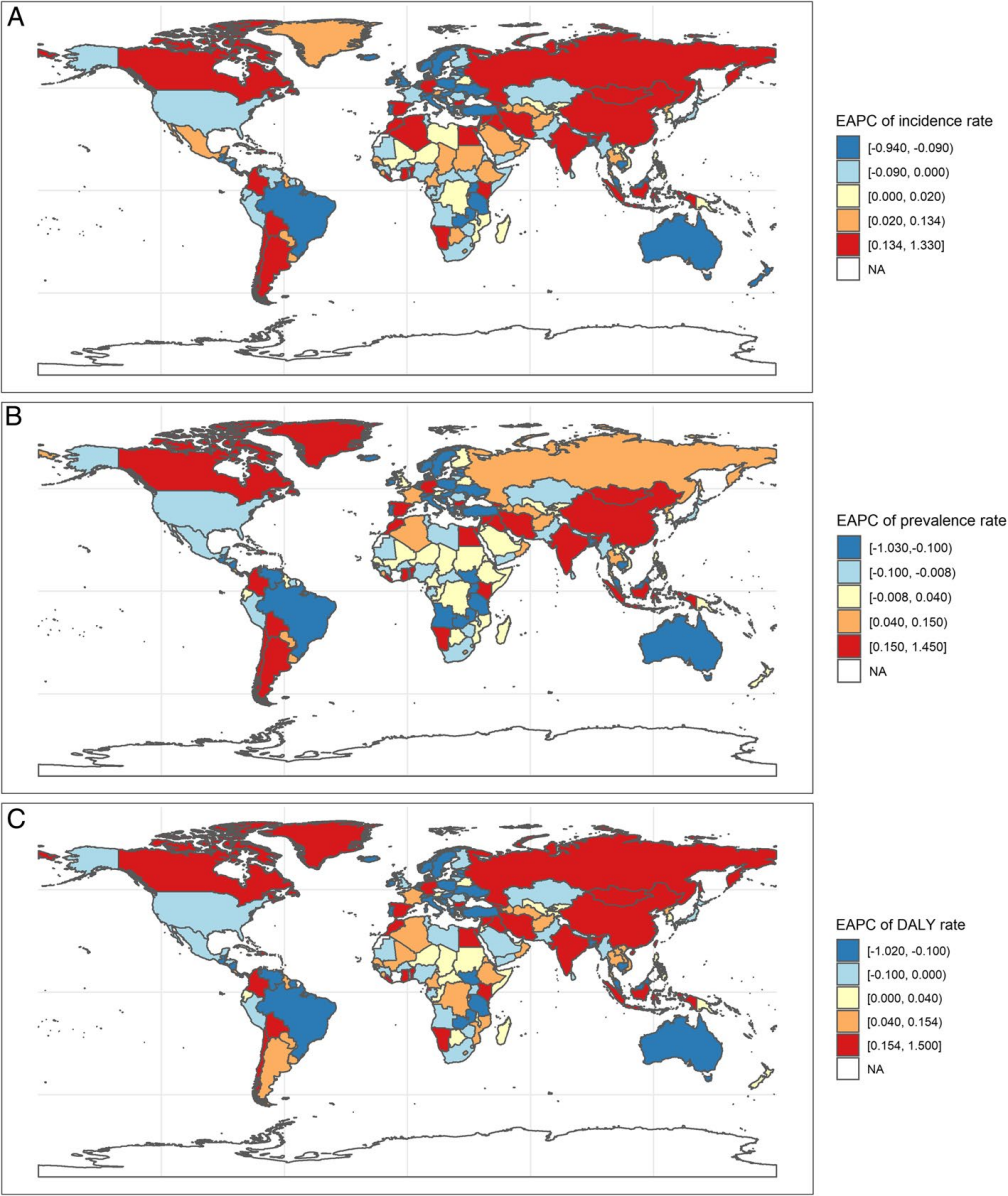
**A** The global EAPC of incidence rate. **B** The global EAPC of prevalence rate. **C** The global EAPC of DALYs rate. EAPC, Estimated Annual Percentage Change; DALYs, Disability-Adjusted Life Years

Over the past 30 years, the countries with the largest increase in ASIR were Kenya and Iran (Islamic republic of), with an EAPC of 1.33% (95% *CI *= 1.06 to 1.60%) and 1.21% (95% *CI *= 0.98 to 1.44%), respectively. In contrast, Australia and Italy have had the most significant declines in ASIR (Supplementary Table 1A, Fig. 2A). Chile and Kenya also had the largest increase in ASPR, with EAPCs of 1.45% (95% *CI *= 1.26 to 1.63%) and 1.41% (95% *CI *= 1.12 to 1.70%), respectively. However, Italy and Australia experienced the greatest decline in ASPR (Supplementary Table 1B, Fig. 2B). The highest EAPCs of ASDR were Chile and Kenya. The decreases were largest in Italy for ASDR, with *EAPCs* =  − 1.02% (95% *CI* =  − 1.25 to − 0.79%) (Supplementary Table 1C). All the ASIR, ASPR, and ASDR changed very little in the five most-populous countries. Positive trends in EAPC of DALYs on the top five ones were Chile (1.50%), Kenya (1.43%), Colombia (1.38%), Iran (Islamic Republic of) (1.38%), and Rwanda (0.87%), while negative trends were Italy (− 1.02%), Australia (− 0.99%), Netherlands (− 0.81%), Bosnia and Herzegovina (− 0.78%), and Dominica (− 0.69%) (Supplementary Table 1C, Fig. 2C).

### The Burden of CUD at the Regional Level

Overall, the numbers of incidence cases, prevalence cases, and DALYs for the 21 regions were mostly increased from 1990 to 2019. However, Australasia, Central Europe, Eastern Europe, Western Europe, and High-income Asia Pacific decreased. Among the 21 analyzed regions, South Asia presented the highest number of incidence cases (1.04 million, 95% *CI* = 0.77 to 1.46 million), prevalence cases (6.10 million, 95% *CI* = 4.47 to 8.23 million), and DALYs (0.18 million, 95% *CI* = 0.10 to 0.27 million) in 2019 (Tables 1 and 2).

The ASIR and ASPR were stable over 30 years in most regions, with only slightly waxing and waning. High-income North America showed the highest ASIR in 2019, at about 149.3/100,000 persons (95% *CI* = 114.7 to 203.8/100,000 persons), while it was lowest in Western Sub-Saharan Africa at 21.9/100,000 persons (95% *CI* = 16.2 to 30.7/100,000 persons). ASPR of these two regions showed the same trend with ASIR. The ASDR in all regions was relatively stable, with only slightly fluctuating. For those five regions that incidence, prevalence cases and DALYs decreased which mentioned above, and the ASDR in these regions showed steady decreased as well. Australasia, Central Europe, Eastern Europe, Western Europe, High-income Asia Pacific, High-income North America, and Southern Sub-Saharan Africa demonstrated negative percentage change in all age DALYs. In addition, the ASDR was highest in High-income North America, at about 28.7/100,000 persons (95% *CI* = 17.4 to 44/100,000 persons) and lowest in Western Sub-Saharan Africa, at about 3.7/100,000 persons (95% *CI* = 2.2 to 5.9/100,000 persons) in 2019.

The highest EAPCs of ASIR and ASPR revealed Southern Latin America, at 0.46% (95% *CI* = 0.38 to 0.55%) and 0.64% (95% *CI* = 0.56 to 0.71%), while the lowest were Australasia, at − 0.80% (95% *CI* =  − 0.96 to − 0.65%) and − 0.81% (95% *CI* =  − 1.01 to − 0.62%), respectively. EAPC of DALYs showed the similar trend with ASIR and ASPR (Tables 1 and 2).

### The Burden of CUD at the SDI-Quintile Level

From 1990 to 2019, the incidence cases and DALYs for high, high-middle, and middle SDI-quintile areas showed stable trends, but in low-middle and low SDI are showed significant growth that almost doubled in incidence cases. In 2019, the incidence cases for the middle SDI and low-middle SDI-quintile areas were becoming higher than the high SDI-quintile region, which was at the top of 1990. The trends of high, high-middle, low-middle, and low SDI-quintile areas in prevalence cases are similar to incidence cases, but the prevalence cases of middle SDI-quintile area increased significantly at 55.5%, from 4.12 million (95% *CI* = 2.94 to 5.61 million) to 6.40 million (95% *CI* = 4.73 to 8.41 million) (Tables 1 and 2).

**Fig. 3:**
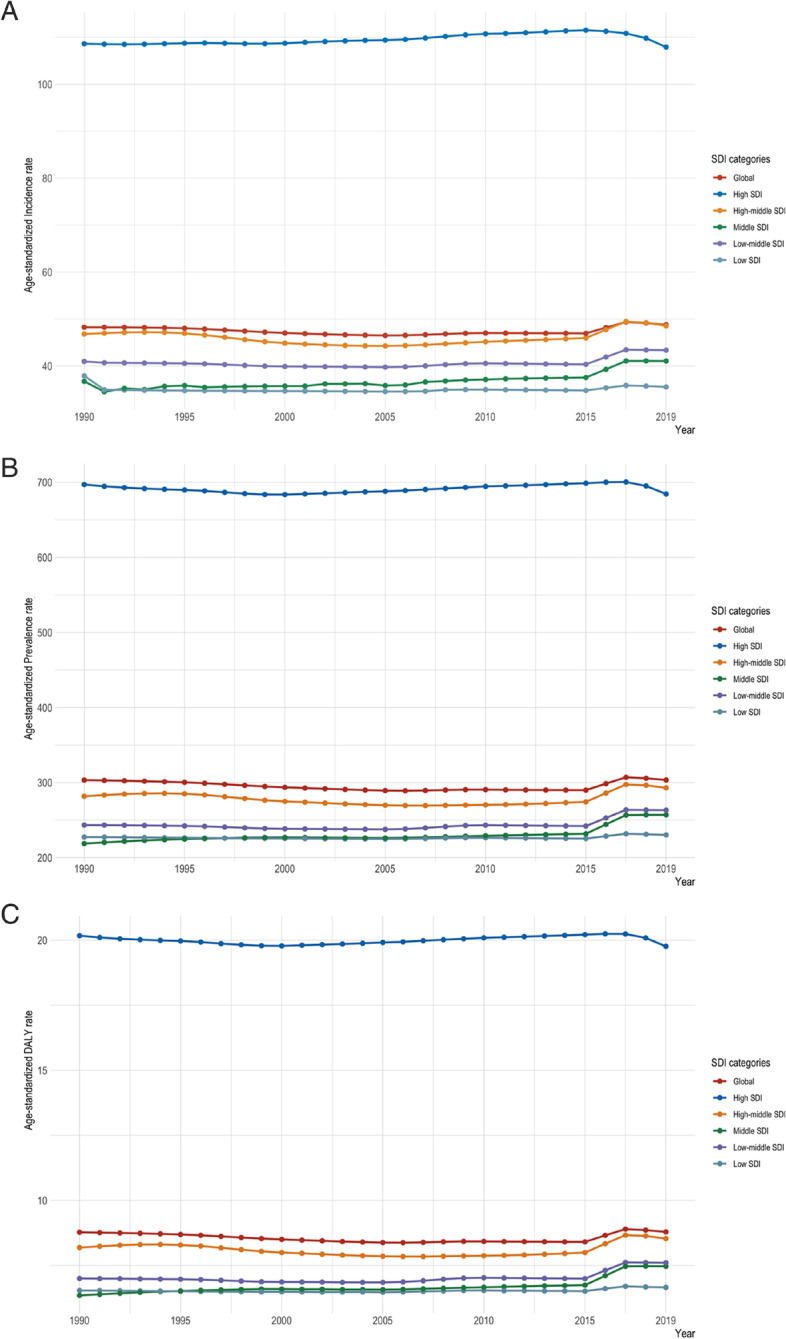
**A** The age-standardized incidence rates (per 100,000 persons) trends of CUD from 1990 to 2019 at SDI quintiles level. **B** The age-standardized prevalence rates (per 100,000 persons) trends of CUD from 1990 to 2019 at SDI quintiles level. **C** The age-standardized DALY rates (per 100,000 persons) trends of CUD from 1990 to 2019 at SDI quintiles level. SDI, Sociodemographic Index; DALYs, Disability-Adjusted Life Years; CUD, Cannabis Use Disorder

Over the past 30 years, the five quintiles of ASIR have been relatively stable, with the highest rate occurring in the high SDI-quintile area at around 107.9/100,000 persons (95% *CI* = 83.6 to 146.5 per 100,000 persons). The rest of the regions had ASIRs ranging from 35.5 to 48.5/100,000 persons (Table 1). What is striking in Fig. 3A is the highest ASIR of the high SDI-quintile area, the rest of the four quintiles were below global values. The trend of ASPR and ASDR indicated the similarity with ASIR (Fig. 3B and C).

## Discussion

This study presents three levels of epidemiological parameters, starting with incidence, prevalence, and DALYs, followed by ASIR, ASPR, and ASDR, and finally, EAPC describes trends over 30 years. Among these, the first-level parameters are affected by the population structure of the corresponding countries and territories, which describes the actual disease status and burden. Second-level parameters facilitate comparisons between countries or regions for CUD burden, as age-standardized methods remove the impact of age structure. Finally, the third-level parameters summarize CUD trends changes over time.

During the past three decades, the global incidence and prevalence cases of CUD have been escalating. In 2019, the incidence cases for males and females were 2.42 million and 1.32 million, respectively. And the prevalence cases were 15.63 million and 8.21 million for males and females. What stands out are the incidence and prevalence of males are nearly double higher than that of females. Some studies indicated males reported using cannabis more frequently and in higher quantities than females. Social factors that limit cannabis exposure and reduce the likelihood of females include more awareness of risk, decreased cannabis use among peers, and greater childcare responsibilities (Cooper & Craft, 2018; Cuttler et al., 2016).

In terms of age, young people aged 20–24 years with CUD had the highest DALYs in 2019, followed by those younger than 20 years old. The high DALYs of adolescents increased the global burden of disease of CUD. Adolescent cannabis users showed a series of deficits such as slower psychomotor speed, poorer attention and memory, and disability in planning and sequencing (Medina et al., 2007). Heavy usage of cannabis during adolescence will cause neurocognitive deficits and functional impairment in the future. The more frequently and began using cannabis at an earlier age experienced worse outcomes and long-lasting effects (Feeney & Kampman, 2016; Meier et al., 2012; Tapert et al., 2008). Adolescent cannabis use will increase the risk of anxiety, depression, schizophrenia, and even increase the possibility of suicide (Gobbi et al., 2019; Malone et al., 2010). This may be closely related to adolescent neuroplasticity and neurocognitive development (Simpson & Magid, 2016). In addition, the 20–24 years old could be regarded as a transitional age group who will be forward from children to adults. It is necessary to pay more attention to the impact of transition-age individuals who use cannabis every day at the second-highest illicit drug except alcohol (Feeney & Kampman, 2016).

India had the highest incidence, prevalence, and DALYs from 1990 to 2019. India, China, and the USA have always been among the top three, which is related to the two largest countries in the world with the population base of India and China. Additionally, the highest DALYs was in the USA in 1990, and in 2019, it shifted to India. India showed the largest increase in DALYs, at 116.8%. The use of cannabis in India has a history of thousands of years and is deeply rooted in Indian legends, religions, and religious rituals (Aroonsrimorakot, 2019). Among them, bhang, charas, or ganja used for religious activities and sacrifices has been passed down to nowadays. According to the National Drug Dependence Treatment Center (NDDTC) report, Indian CUD is second only to alcohol abuse, about 2.8% of the population (31 million individuals) reported having used any cannabis product within the previous year (National Drug Dependence Treatment Centre & All India Institute of Medical Sciences, 2019). Its popularity also showed a shocking degree and the epidemic of CUD in the young generation has also assumed alarming dimensions. However, different states have their own laws regarding the consumption, possession, sale, or purchase of cannabis (Dube & Dhingra, 2020). Many of the above reasons may have affected India’s very high incidence, prevalence, and DALYs of CUD.

In 2019, the highest ASDR and ASPR were in Canada and the USA, respectively. The legalization of commercial cannabis production for medicinal and recreational purposes in North America may change the global cannabis market (Hall et al., 2019). Legalization and decriminalization of cannabis may increase the regular use of cannabis users and, in the long term, increase the harm associated with use, because it will make more effective cannabis products cheaper and more readily available. After the legalization of cannabis in some states in the USA, the numbers of users, combined drug overdose, and the hospital admission rate have also gradually increased, which has increased the burden of disease to a certain extent (Zvonarev et al., 2019). However, some studies revealed that as the use of medical cannabis increases, the number of opioid prescriptions, dependence, and the number of opioid poisoning patients who are receiving opioid treatment have declined (Bachhuber et al., 2014; Bradford et al., 2018; Liang et al., 2018; Wen & Hockenberry, 2018).

Over the past three decades, Qatar has dramatically increased 538.7% in incidence and a 656.3% increase in prevalence. Although Qatar has a small size of population, as one of the countries with the highest per capita GDP, it attracts people from all over the world to work and live in this country (De Bel-air, 2014). Between 2007 and 2017, substance use disorders in Qatar rose from the third to the top cause of disability. The government has transformed addiction from a criminal issue to a public health issue, trying to legislate to improve and reduce substance abuse (Alabdulla et al., 2022). Kenya has high EAPC of ASIR, ASPR, and ASDR due to the country has complex historical, political, and economic origins of cannabis abuse (Ndanyi, 2021). Poverty, political instability, social unrest, and refugee issues in Africa have led to the rapid spread of psychoactive substance use, especially among young people. Low educational attainment is a risk factor for cannabis use disorder (Ngarachu et al., 2022; Odejide, 2006).

Our results indicate that the incidence cases, prevalence cases, and DALYs of CUD in 21 regions of the world have generally increased, but Australasia, Europe, and High-income Asia Pacific have declined. High-income North America had the highest ASIR in 2019, while sub-Saharan Africa had the lowest. Australasia, Western Europe, Central Europe, Eastern Europe, High-income Asia Pacific, High-income North America, and Southern Sub-Saharan Africa indicated negative percentage change in all age DALYs. According to a report released by UNODC in 2020, there were an estimated 192 million cannabis users worldwide in 2018, equivalent to 3.9% of the global population aged 15–64. Cannabis use in the past year was significantly higher than the global averages in North America (14.6%), Australia and New Zealand (10.6%), and West and Central Africa (9.3%). The decline in the incidence and prevalence of cases in the appealed regions may be due to the increase in the number of cannabis users worldwide, while these regions have remained stable, and statistics have shown a relative decline (United Nations Office on Drugs & Crime, 2020c).

From 1990 to 2019, the incidence of cases and DALYs in the high, middle, and middle SDI-quintile demonstrated a stable trend, but the incidence of cases and DALYs in the low-middle and low SDI areas indicated a significant increase, and the incidence of cases almost double change. Reports revealed that the higher socioeconomic classes had higher annual prevalence of drug use, while the lower socioeconomic classes had higher rates of drug dependence. People living on the margins of society are often more likely to switch from recreational drug use to full-scale drug abuse and drug dependence because treatment facilities that intervene in the early stages of the drug occupation are often unaffordable (United Nations Office on Drugs & Crime, 2020a). The prevalence of high, middle, low, middle, and low SDI-quintile showed similar trends as the incidence of cases, but the mid-SDI-quintile areas increased significantly, with an increase of 55.5%. The ASIR, ASPR, and ASDR in the high SDI quintile areas are the only areas much higher than the global average, and the remaining quartile areas are all lower than the global average. The dynamics driving the current global drug market expansion and increasing complexity are multifaceted, including demand-driven, supply-driven, and control-driven (United Nations Office on Drugs & Crime, 2020a). However, among adults living in high-income countries, drug abuse disorders are often more common in socioeconomically disadvantaged groups (United Nations Office on Drugs & Crime, 2020d).

## Limitation

There are some limitations to our study. First, data gaps, variable data quality, and uncertainty after modeling with these data will overestimate or underestimate the disease burden of CUD. For example, an important change in the estimated cause of death that restricts the use of all substances is the ICD code. Some countries have additional codes that allow more accurate causes of death to be attributed to specific substances, which leads to data uncertainty. Second, public health and data collection in some countries are relatively weak, and it is doubtful whether CUDs of disease burden are accurately reflected. Third, GBD uses the ICD-10 system to classify injuries and diseases. The introduction of DSM-V by the American Psychiatric Association included a shift from DSM-IV abuse and dependence to a category of use disorder, defined as mild, moderate, and severe in severity. Moderate to severe substance use disorders in DSM-V may be higher than DSM-IV and ICD-10 dependence, which means that if DSM-V prevalence estimates are used, the estimated burden of substance use disorders may be higher (Pan et al., 2020). Fourth, more and more evidence showed that there is a causal relationship between cannabis use and traffic accidents, and whether accidental injuries are included in the disease burden of CUD is still controversial.

Furthermore, we observed inconsistencies from similar studies. Besides the above limitations, we also found examples of significant differences in findings between regional studies and the GBD database. Based on the data from the GBD database, we can conclude that the incidence of CUD, prevalence, and DALYs in the European region have declined over the last three decades. However, the study by *Manthey* et al. expressed the fact that cannabis use and related problems in Europe, estimated using data from the European Monitoring Center for Drugs and Drug Addiction (EMCDDA) survey, have increased over the last decade (Manthey et al., 2021). The prevalence of CUD on GBD data is estimated and adjusted based on any type of cannabis use including originally reported cannabis use, regular (ie. weekly) cannabis use, and cannabis dependence. The different trends between two research have questioned the CUD estimates. This may be due to the fact that CUD data from statistical models is smoothed estimates and that GBD input data has some inherent problems. Some studies (Manthey & Rehm, 2019; Xia & Huan, 2017) have also questioned the consistency of selected GBD estimates, and therefore, the CUD prevalence estimation procedures need to be revisited and improved. Another consideration is that the validity of CUD estimates may vary from country to country, which limits the comparability of estimates across countries.

## Conclusion

Nearly 200 million individuals are cannabis users worldwide, and CUD is a notable risk factor for the global burden of diseases. Especially, males have a higher incidence and prevalence of cases, whereas females should not be underestimated. The global cultivation of cannabis, rooted in different cultures, diversified access to cannabis, legalization in controversy, the promotion of medical cannabis, and many other factors promote the global cannabis industry is constantly updated and upgraded. Adolescents’ exposure to cannabis during the transitional period leads to a range of neuropsychiatric sequelae, neurodevelopmental disorders, and varying degrees of anxiety, depression, and cognitive dysfunction appearing in adults with CUD, exacerbating the disease burden of CUD. With the pandemic of COVID-19, lifestyles have undergone fundamental changes and the world economic landscape has undergone a complex and profound transformation as well. CUD still deserves more discussion in the future in terms of pathophysiological mechanisms, socioeconomics, law, and policy improvements. Even though the CUD estimates from GBD did not parallel survey-derived prevalence estimates of cannabis use, weakening the confidence in the GBD data, this does not detract from the fact that the GBD database is still the most consistent source of data to reveal the burden of certain kind of disease in global level.

## Supplementary Information

Below is the link to the electronic supplementary material.

Supplementary file1 (XLSX 47 KB)

Supplementary file2 (XLSX 47 KB)

Supplementary file3 (XLSX 43 KB)

Supplementary file4 (XLSX 16 KB)

Supplementary file5 (XLSX 16 KB)

Supplementary file6 (XLSX 16 KB)

## Data Availability

The raw data for this research were from the GBD online data source tool, which is located at https://vizhub.healthdata.org/gbd-results/.
